# Processing of Whole Kernel Tapioca Pearl and Milk Tea BOBA of Fresh Highland Barley: Optimization of Processing Parameters and Quality Evaluation

**DOI:** 10.3390/foods13060927

**Published:** 2024-03-19

**Authors:** Jiawen Zhu, Jiayao Li, Huajun Wu, Yingying Zhu, Jilin Dong, Rongjie Huang, Ruiling Shen

**Affiliations:** 1 College of Food and Bioengineering, Zhengzhou University of Light Industry, Zhengzhou 450002, China; m18813298282@163.com (J.Z.); zhuying881020@163.com (Y.Z.); djl1968@163.com (J.D.); nysyhrj@163.com (R.H.); 2 Food Laboratory of Zhongyuan, Luohe 462300, China; 3 Henan Key Laboratory of Cold Chain Food Quality and Safety Control, Zhengzhou University of Light Industry, Zhengzhou 450002, China

**Keywords:** fresh highland barley, heat-treated, tapioca pearl, milk tea BOBA

## Abstract

Fresh highland barley is difficult to store, leading to a lack of commercial products. To address these problems, the research investigated the effect of different heat treatments (steaming <SFB>, microwaving <MFB>, baking <BFB>, and cooking <CFB>) on the quality of fresh highland barley, and used pretreated fresh highland barley as material, combined with the milk tea market, to design and optimize the preparation process of fresh highland barley tapioca pearl and milk tea BOBA. The results showed that the different heat treatments reduced the content of ash and starch significantly, and SFB and MFB decreased the digestibility of fresh highland barley (P < 0.05). In particular, SFB had a significantly higher overall score for fresh barley than the other treatments, with the highest sensory evaluation for aroma, elasticity, and the overall taste of the grain, and the eGI value was the lowest (58.64). The optimal preparation process of fresh highland barley tapioca pearl and milk tea BOBA was designed and optimized by the L_9_(34) orthogonal test. The optimal tapioca pearl formula contained the following: apioca starch content of 36%, cooking time of 2.5 min, and erythritol stevia content of 1.5%. The optimal milk tea BOBA formula contained the following: sodium alginate content of 1.3%, erythritol stevia content of 0.6%, and calcium lactate content of 2.2%. This not only improves the comprehensive utilization rate of fresh highland barley, but also provides the accessory food, ensuring a lower eGI and increasing the healthiness and diversity of milk tea.

## 1. Introduction

Highland barley is the main crop in the Qinghai-Tibet region, which is rich in β-glucan, γ-aminobutyric acid, and other functionally active components [1]. Fresh highland barley is the barley which is harvested from late lactation to early waxing [2], and according to the literature, compared with mature barley, fresh highland barley contains 55~65% water, 12~14% protein, and about 13% dietary fiber [3], while the starch content only accounts for about 50% of the dry base material of barley, which is ideal for diabetic patients’ food [4]. Bueno et al. [5] reported that the use of the green wheat decreased body weight percentage, visceral fat, glycemia, low-density lipoprotein cholesterol, triglycerides, and atherogenic indices. Thus, it can be seen that fresh highland barley has higher health benefits, and the promotion of its consumption and the development of its deep processing technology and products will gradually become a new research hotspot.

However, as a seasonal food, fresh highland barley is suitable for harvesting, processing, and consumption in less than 10 days. Various enzymes such as polyphenol oxidase (PPO), peroxidase (POD), and lipase (LA) are still active after harvesting [6], which not only cause enzymatic browning and off-flavors, but also consume their own nutrients and deteriorate the taste [7]. At present, the method of freezing at −18 °C is commonly used to reduce the quality deterioration of fresh highland barley due to respiratory depletion during storage, transportation, and processing. However, this method not only fails to solve the fundamental problem, but also greatly increases the energy consumption and the costs incurred during the transportation and storage of raw materials. Therefore, it is necessary to optimize the stabilizing treatment of fresh barley and develop new products with high sales volumes and broad market prospects. Zhang and Song et al. [8] used superheated steam, microwaves, and hot water rinsing methods to sterilize green barley kernels with blunt enzymes, which effectively prolonged the shelf life of green barley kernels. Li Jianli et al. [9] studied the effects of different drying methods on the nutritional composition and physicochemical properties of green barley kernels and determined the optimal drying process.

At present, the green wheat has been used to make cakes, noodles, bread, fried dough sticks, steamed buns, Nianzhuan, Zongzi, health drinks, etc., as a food raw material [10,11]. However, the seasonal limitation of fresh highland barley not only affects the storage quality of the barley itself, but also has a significant impact on the production and processing afterwards. Zhang Kang Yi [12] prepared composite nutritional flour from green wheat kernels, corn, and green beans, and then determined the optimal spray drying process conditions using response surface tests. The team also developed high-quality noodles, cookies, steamed buns, and bread by compounding green wheat kernel flour with wheat flour or gluten flour with different gluten characteristics in a certain ratio. Nianzhuan is a traditional Chinese food made by squeezing green wheat kernels, and Jin Yadong et al. [13] determined the optimal processing conditions by improving the conditions for the traditional processing of Nianzhuan. Roasted green wheat is a traditional food produced in the Middle East that is made by roasting and kneading green wheat kernels, and it is usually exported to Europe. However, in general, the production of fresh highland barley is still in the mode of small-scale farm production, lacking industrialized production on a massive basis, and there is virtually no commercial market of any form of fresh highland grain for sale.

The milk tea ingredient, as solid ingredients in milk tea beverages, add a chewy experience to milk tea and give it a richer taste. A variety of grains have been used in the development of milk tea ingredients, such as oats, barley, black rice, and mature barley, but fresh grains have not been seen in milk tea ingredients. At present, the main forms of milk tea ingredients are whole kernel, tapioca pearl, and milk tea BOBA, regardless of the form of the product, and they contain a large amount of sugar, resulting in a high calorie content and low nutritional value, which cannot meet the consumer demand for healthy milk tea products. Fresh highland barley has higher health benefits and a lower GI value, combined with the milk tea market, and it will be more popular among contemporary young people.

The aim of this work was to protect the color of fresh highland barley and stabilize it by different heat treatments to optimize the best pretreatment method, and finally combine it with the milk tea market and developed products, which are popular among contemporary young people, by using the pretreated fresh highland barley, namely fresh highland barley tapioca pearl and milk tea BOBA.

## 2. Materials and Methods

### 2.1. Material

The fresh highland barley was provided as grains, which were harvested in one month before maturity by the Qinghai Provincial Light Industry Research Institute Co., Ltd., Xining, China. Total starch assay, glucose oxidase/peroxidase (GOPOD), dietary fiber, and β-glucan assay kits were provided by Megazyme International Ireland Ltd. (Bray, Ireland). Alpha-amylase (EC 3.2.1.1), amyloglucosidase (EC 3.2.1.3), pepsin (EC 3.4.23.1), and trypsin (EC 3.4.4.4) were purchased from Sigma Chemical Co. (St. Louis, MO, USA). All other chemicals were of reagent grade unless otherwise stated.

### 2.2. Color Protection of Fresh Highland Barley

We followed the conditions obtained from previous optimal experiments (unpublished data). We chose fresh highland barleys which were reaped in the late milk-ripening stage of the barley-growing period, removed the shell after freezing, washed the grains of fresh highland barley with distilled water 3 times, soaked for 30 s with 30 g/L of salt solution to remove impurities, and then took them out immediately and soaked for 30 min with distilled water. Finally, the water was filtered and the fresh highland barley was preserved, waiting for the next step.

### 2.3. Thermal Processing of Fresh Highland Barley

First, 100 g of fresh highland barley was heated by steaming (SFB), microwave (MFB), baking (BFB), and cooking (CFB), respectively, according to the conditions obtained from previous optimal experiments (unpublished data). The specific method was as follows: the fresh highland barley was laid in a tray placed into the microwave oven (Midea, M1-L236C, Foshan, Guangdong, China) and microwaved at 400 W for 30 s; or on the tray of the baking oven (Midea, MG38CB-AA, Foshan, Guangdong, China) and baked at 100 °C for 15 min; or the fresh highland barley was laid in a thin layer of 0.5 cm on the grate of the electric steamer (Fotile, SCD20-01, Ningbo, China) and steamed at 103 °C for 15 min under atmospheric pressure; or the fresh highland barley was put under boiling water (100 °C) and boiled for 10 min. After these treatments, all the samples were cooled to room temperature and stored in a 4 °C refrigerator for testing. Meanwhile, a blank control group was set up without heating treatment (UT) and maturated highland barley.

### 2.4. The Effect of Different Thermal Treatments for Fresh Highland Barley

#### 2.4.1. Nutrient Composition Analysis

The ash, crude fat, and crude protein contents of samples were determined using GB 5009.4-2016 method [14], GB 5009.6-2016 method [15], and GB 5009.5-2016 method [16], respectively. The total starch contents were determined using AOAC 996.11. The total dietary fibers (TDF) contents were determined using the dietary fiber kit (Megazyme International, Bray, Ireland) following the approved AOAC 985.29[18].

#### 2.4.2. Color Analysis

The color measurements of the heat-treatment fresh highland barley samples’ upper and bottom surfaces (8 mm Ø contact area) were carried out in triplicates using a colorimeter (Ci7600, Aiseli, Grand Rapids, MI, USA). The instrument was calibrated using a standard light white reference tile and the measurements were performed under standard illuminant D65. The obtained results were expressed in terms of L* (lightness), a* (redness to greenness), and b* (yellowness to blueness) values.

#### 2.4.3. In Vitro Starch Digestion

The extent of in vitro starch hydrolysis was assessed using the method published by Huang Lu et al. [14], with a few adjustments. First, 10 mL of deionized water was added to the samples (which is 1 g of starch equivalent blended on a dry weight basis), which was then combined with 5 mL of pepsin solution at 37 °C with constant stirring for 30 min. Then, 5 mL of simulate intestinal fluid was injected and the volume was fixed to 50 mL. The entire digestion process was constant at 37 °C. Take out the required samples at each time point and 0.5 mL of the samples was taken at 0, 10, 20, 30, 45, 60, 90, 120, 150, and 180 min. The enzyme was deactivated by 4.5 mL of anhydrous ethanol and centrifuged at 3000 r/min for 5 min. After centrifugation, the 2,4-dinitrosalicyclic acid (DNS) assay was used to assess the amount of reducing sugars in the supernatant. The starch digestibility was calculated according to the following Formula (1):

(1) $$\mathrm{Hydrolysisrate}\;(\%)=\frac{reducing\;sugar\;content}{total\;starch}\;\times \;100\%$$

The eGI value was calculated according to the following Formula (2):

eGI = 39.71 + 0.549HI(2)

where eGI was the expected glycemic index and HI was the Hydrolysis index.

#### 2.4.4. Sensory Descriptive Analysis 

The sensory analysis method using Aleksandra and Dubravka [15] with some modifications was used. A well-trained panel, consisting of 10 women and 10 men, aged from 20 to 50 years, evaluated the samples randomly in a sensory evaluation room at room temperature. Initially, a list of relevant sensory attributes was discussed and agreed upon by the assessors, along with a short and precise definition of each attribute (Table 1). The following descriptors were assessed: the appearance, aroma, mouth feel, and taste were rated from very unattractive (1) to very attractive (5). Water was provided for mouth rinsing between the evaluations of different samples to avoid the carryover effect of the aftertaste.

#### 2.4.5. Texture Profile Analysis (TPA)

The texture of the heat-treatment fresh highland barley was determined by TA. (TA. New Plus, Isenso, NY, USA) with piston P/36R. The compression distance was adjusted as 10 mm. The time between the tow compression was 5.0 s, and the trigger force was 5 g. The pre-test, test, and post-test speeds were fixed at 1.00 mm/s, 2.00 mm/s, and 2.00 mm/s, respectively. Each determination was carried out 10 times.

### 2.5. Food Product-Making and Optimizing

#### 2.5.1. Tapioca Pearl-Making Process

Add erythritol stevia, sodium diacetate, sodium dehydroacetate, and hydroxypropyl di-starch phosphate to distilled water in turn and place them in a boiling water bath to stir constantly. Stop heating immediately and stir quickly after adding tapioca starch. Roll into a dough shape after stirring evenly. Brush the dough surface with a layer of tapioca flour and then cut into small pieces of 5 mm. Add pre-treated fresh highland barley into the small dough and knead into a pearl shape. Put them into boiling water until boiled.

#### 2.5.2. Milk Tea BOBA-Making Process

Put the erythritol stevia, 0.39% D-isoascorbic acid sodium, and 0.3% vitamin C in distilled water, respectively. Heat the solution until boiling. Prepare sugar water by continuously stirring. Put the sodium alginate in the sugar water and then add the SFB after mixing them uniformly, then put them one by one into a 2% calcium lactate solution, soak for a period of time, take them out, filter off the excess water, and obtain the barley BOBA of milk tea.

#### 2.5.3. Experimental Design for Optimization

Based on the previous results of single-factor experiments, the optimal preparation process of fresh highland barley tapioca pearl and milk tea BOBA was designed and optimized by the L_9_(3^3^) orthogonal test. For the preparation process of tapioca pearl, three variables, the concentration of tapioca starch and erythritol stevia, and the time of cooking, were selected for further optimization. As shown in Table 2, for three factors at three levels each, the orthogonal test was carried out in nine experiments, which were carried out using a tapioca starch concentration of 34%, 36%, and 38%, erythritol stevia concentration of 1.5%, 2%, and 2.5%, and cooking time of 1.5, 2, and 2.5 min, and all the experiments were carried out in triplicate. For the preparation process of milk tea BOBA, three variables, the concentration of sodium alginate, calcium lactate, and erythritol stevia, were designed and optimized by an orthogonal test. For three factors at three levels each, the orthogonal test was carried out in nine experiments, which were carried out using a sodium alginate concentration of 1.1%, 1.3%, and 1.5%, calcium lactate concentration of 0.4%, 0.5%, and 0.6%, and erythritol stevia concentration of 1.8%, 2.0%, and 2.2%, and all the experiments were carried out in triplicate. 

#### 2.5.4. Sensory Evaluate

The sensory evaluate was assessed using the method published by Aleksandra Torbica [16], with a few adjustments. In order to optimize the preparation process and gain the optimal formula of tapioca pearl and milk tea BOBA, it was conducted by the same group with 1.4.4. The panelists were asked to evaluate each product attribute applying a hedonic scale of 100 points (10—dislike very much, 100—like very much). In order to evaluate tapioca pearl quality, the following attributes were examined: appearance, taste, color, and mouth feel. Appearance was assessed in terms of size, surface diaphaneity, adhesiveness, and the existence of cracks. Taste was defined as the existence of aromas typical of cereals and tapioca pearl. Color was assessed in terms of the brightness and the uniformity of green. Mouth feel was evaluated as cohesiveness and elasticity. In order to evaluate milk tea BOBA, the following attributes were examined: appearance, taste, color, and mouth feel. Appearance was assessed in terms of size uniformity and overall completeness. Taste was defined as the existence of aromas typical of cereals and milk tea BOBA. Color was assessed in terms of the brightness and uniformity of green. Mouth feel was evaluated as the breakdown force which appeared at the first chew.

### 2.6. Statistical Analysis

Data were expressed as the mean ± SD, and included at least 3 replicates per sample. The Origin Pro 21 program (Origin Lab Inc., MA, USA) was used to organize and draw the data. One-Way Analysis of Variance (ANOVA) and Tukey’s test were performed using SPSS (version 20.0, Chicago, NY, USA). The statistical significance was set at *p* < 0.05.

## 3. Results

### 3.1. The Effects of Different Treatments on the Nutrient Composition of Fresh Highland Barley

The effects of the different heat treatment methods on the nutrients of fresh highland barley are shown in Table 3. Compared with CON, the ash and starch content of fresh highland barley was significantly lower, while the lipid, protein, and dietary fiber contents were significantly higher than those of mature highland barley, which is due to the continuous decomposition of nutrients such as protein and dietary fiber during the ripening process of the grain. The lower starch content is further evidence that fresh highland barley is ideal for diabetic patients’ food [4]. In addition, the ash and starch contents were significantly lower (*p* < 0.05) and the protein content increased in the four heat-treated fresh barley compared with the UT group, the lipid content of baked and microwaved fresh highland barley was not significantly different, while steamed and cooked decreased (*p* < 0.05), and there was no significant change in dietary fiber. This may be due to some minerals being easily volatilized during heating (e.g., iodine, selenium, etc.) [17], starch being decomposed into dextrin or reducing sugar [14], and fat being decomposed into fatty acids, monoglyceride fat, and compounded with starch or protein. The results showed that compared with mature highland barley, the nutrient content of fresh highland barley is higher and more beneficial to human absorption, and different heat treatments can affect the basic nutrients, especially reducing the content of ash and starch.

### 3.2. The Effects of Different Treatments on the Color of Fresh Highland Barley

Color is an essential characteristic of food sensory as it influences consumer acceptance along with texture and taste [18]. The color depends on the characteristics of the food and the processing conditions. The results of the study on the color of fresh barley with different heat treatments are shown in Table 4. Compared with the control group, the difference in the brightness of steamed fresh barley grains was not significant (*p* < 0.05), while the other three groups were significantly lower. In particular, the L* of fresh barley in the baking treatment decreased from 80.04 to 75.47, which may be caused by the degradation of color-presenting substances during the overheating process [19]. Fresh barley was in full contact with the oxygen in the oven, resulting in the oxidative browning of green wheat kernel and reduced brightness [20]. In contrast, no significant difference occurred for a* and b*, except cooked-treat, indicating that heat treatment can effectively maintain the color of fresh barley, probably because heat treatment destroys the activity of chlorophyll degradation-related enzymes and reduces the deenzymatic degradation of chlorophyll, thus providing color protection [21].

### 3.3. The Effects of Different Treatments on the Sensory of Fresh Highland Barley

**Figure 1:**
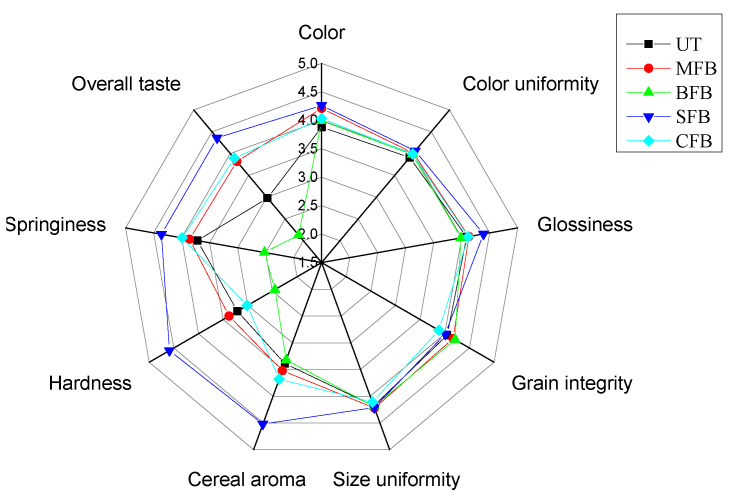
Sensory analysis of fresh highland barley with different treatments (UT, MFB, BFB, SFB, and CFB are untreated, microwaved, baked, steamed, and cooked fresh highland barley, respectively).

The effects of different heat treatments on the sensory characteristics and overall acceptability of fresh barley are shown in Figure 1. The panel concluded that the different heat treatments did not significantly affect the appearance of fresh barley, with scores higher than four for color, color uniformity, glossiness, grain integrity, and uniformity. The taste and overall flavor of fresh barley from the baking treatment were significantly lower than those from the other treatments, probably because the aroma components were severely damaged and a larger amount of water was lost during the baking process, resulting in dry and hard barley grains and a poor taste and overall flavor. Steaming contributed to a significantly higher overall score for fresh barley than the other treatments, with the highest scores for aroma, elasticity, and the overall taste of the grain. This was due to the steaming process minimizing damage to grain integrity and aroma and improving barley texture due to the pre-cured process, resulting in a more elastic and uniform color.

### 3.4. The Effects of Different Treatments on the TPA of Fresh Highland Barley

The sensory evaluation and the quality data in Table 5 indicate that the microwave fresh barley has a higher sensory score, moderate elasticity, and viscosity, but the hardness and chewiness of the kernels are higher, which leads to poor taste; cooking fresh barley has moderate hardness and elasticity but poor chewiness; baking fresh barley has poor elasticity, and its hardness and viscosity are obviously higher, which results in poor taste quality; and steaming fresh barley has the best elasticity and moderate hardness and chewiness. In summary, the sensory evaluation and textural characteristics of fresh barley steamed at 103 °C for 20 min were the best and could be used for fresh barley pre-cured treatment.

### 3.5. The Effects of Different Treatments on the Digestibility In Vitro of Fresh Highland Barley

**Figure 2:**
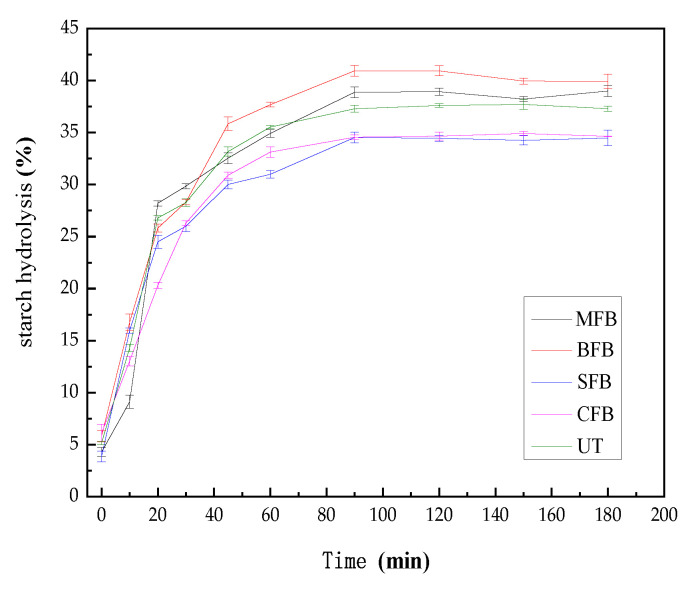
In vitro starch digestibility of fresh highland barley with different treatments (UT, MFB, BFB, SFB, and CFB are untreated, microwaved, baked, steamed, and cooked fresh highland barley, respectively).

The in vitro starch digestibility of fresh barley heat-treated in different ways is shown in Figure 2. From Figure 2, it can be observed that the in vitro starch hydrolysis rate of fresh barley increased rapidly from 0 to 30 min, slowed down from 30 to 90 min, and leveled off after 90 min until it ended after 180 min. The microwave and baking treatments increased the starch hydrolysis rate compared to the untreated group control, and this result is consistent with the findings of Ke et al. [22]. The reason for this phenomenon may be due to the fact that the structural breakdown of starch caused by dry heat treatments such as baking leads to an increase in starch digestibility [14]. In contrast, as Table 6 shows, the steaming and cooking treatments caused a significant decrease in the in vitro starch digestibility of fresh barley. In addition, the eGI value of the steaming and cooking treatments was lower than that of other groups for 58.64 and 58.72. This is because during the steam and cooking treatments, the interaction between the starch chains inside the crystal structure is enhanced, and new amorphous structures are formed, which promote the interaction between amylose and amylopectin within the starch chains and prevent amylase from entering the starch chains. Thus, this leads to an increase in the resistance of starch to enzymatic hydrolysis and a decrease in the digestibility of starch. As a result, the steam and cooking treatments can regulate the in vitro starch digestibility of fresh barley. Kang et al. [23] compared the changes in wheat starch-lauric acid complexes under dry heat (baking, microwaving) and moist heat (steaming, cooking) and found that the digestibility of starch-lauric acid complexes was higher in the dry heat treatment than in the moist heat treatment. This is due to the fact that the higher thermal energy in the dry heat treatment disrupts the crystal structure of the wheat starch-lauric acid complex, which increases the digestibility.

### 3.6. Tapioca Pearl Formula Optimization

**Figure 3:**
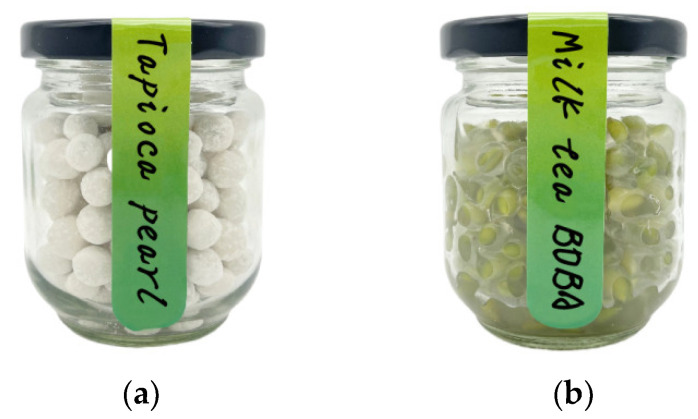
The product of fresh highland barley. (**a**): Tapioca pearl, (**b**): milk tea BOBA.

The product of the tapioca pearl of fresh highland barley is shown in Figure 3a, and the result of the orthogonal test of the tapioca pearl is shown in Table 7. The results of the extreme difference showed that the influential order of the three factors on the tapioca pearl sensory score was tapioca starch content (R = 5.33) > cooking time (R = 5.0) > erythritol stevia content (R = 1.67) (Table 7). The results were consistent with the values of F and P in variance analysis (Table 8). According to the analysis of variance, the effects of tapioca starch content (*p* < 0.01) and cooking time (*p* < 0.01) on the sensory score of tapioca pearl were highly significant, and erythritol stevia content (*p* < 0.05) was significant. Based on extreme difference analysis and analysis of variance, the optimal tapioca pearl formula contained the following: tapioca starch content of 36%, cooking time of 2.5 min, and erythritol stevia content of 1.5%. As shown in Figure 3a, the use of fresh highland barley raises the level of dietary fiber and enhances the health advantages of the tapioca pearl, which are made in accordance with the formula, and after boiling it will have good flexibility, a crystal clear, delicate taste, and a consistent texture.

### 3.7. Milk Tea BOBA Formula Optimization

The product of milk tea BOBA of fresh highland barley is shown in Figure 3b, and the result of the orthogonal test of milk tea BOBA is shown in Table 9. The results of the extreme difference showed that the influential order of the three factors on the milk tea BOBA sensory score was sodium alginate content (R = 8) > erythritol stevia content (R = 5.33) > calcium lactate content (R = 4) (Table 9). The results were consistent with the values of F and P in variance analysis (Table 10). According to the analysis of variance, the effects of sodium alginate content on the sensory score of milk tea BOBA were highly significant (*p* < 0.01), and the erythritol stevia content and calcium lactate content were significant (*p* < 0.05). According to the extreme difference analysis and variance analysis, the optimal milk tea BOBA formula contained the following: sodium alginate content of 1.3%, erythritol stevia content of 0.6%, and calcium lactate content of 2.2%. Sodium alginate is widely used as a membrane material due to its safety and non-toxicity, great film-forming properties, and biocompatibility. Calcium lactate increases the calcium content and coagulability, making the milk tea BOBA easier to mold. Erythritol stevia, as a complex sugar substitute without sugar and calories, is stress-free for a healthy diet. The use of fresh highland barley and the sugar alternative in this formula not only helps to reduce the health risks caused by high sugar intake, but also add healthy and tasty nutritious contents to milk tea. These findings contribute to improving the utilization and development of fresh grains while allowing people to enjoy milk tea BOBA with a low sugar intake and obesity risk.

## 4. Conclusions

In conclusion, the nutritional composition, color, sensory, texture, and digestibility of fresh highland barley were modified by BFB, SFB, MFB, and CFB. The results showed that compared with mature highland barley, the nutrient content of fresh highland barley is higher and more beneficial to human absorption, but compared with fresh highland barley, the different heat treatments can affect the basic nutrients, especially reducing the content of ash and starch. SFB and MFB decreased the digestibility of fresh highland barley compared with UT, but CFB and BFB increased the digestibility of fresh highland barley. All heat treatments significantly changed the sensory, color, and texture of barley (*p* < 0.05). In particular, SFB has the best elasticity and moderate hardness and chewiness, and has a significantly higher overall score for fresh barley than the other treatments, with the highest sensory evaluation for aroma, elasticity, and the overall taste of the grain. As a result, choose the fresh highland barley stream-treated to create a commercial product. The optimal preparation process of fresh highland barley tapioca pearl and milk tea BOBA was designed and optimized by the L_9_(3^3^) orthogonal test. The optimal tapioca pearl formula contained the following: apioca starch content of 36%, cooking time of 2.5 min, and erythritol stevia content of 1.5%. The optimal milk tea BOBA formula contained the following: sodium alginate content of 1.3%, erythritol stevia content of 0.6%, and calcium lactate content of 2.2%.

Milk tea is becoming increasingly popular as a beverage all around the world. We can assume that this trend will result in milk tea and its ingredients’ popularity increasing rather than decreasing. Instead of banning young people from drinking milk tea, we should identify healthier ingredients for this beverage, such as whole kernels. In this sense, our research offers a crucial insight into how to make nutritious tapioca pearls and milk tea BOBA made from fresh highland barley. This research discovery would not only enrich the type of fresh highland barley products, but also provide more healthy nutrients in milk tea.

## 5. Patents

Jilin Dong, Chi Ming, Yingying Zhu, Jiawen Zhu. Fresh highland barley hulling screening and color protection integrated device [P]. Henan: ZL 2020 2 2719524.X.

## Figures and Tables

**Table 1:** Sensory attributes with definitions and end anchors of different heat-treatment fresh highland barley.

Sensory Attributes	Descriptor	Definition with End Anchors
Appearance	Color	Degree of dominant color (light—much)
	Color uniformity	Evenness of color on the sample surface (uneven/nonuniiform—even/uniform)
	Glossiness	Amount of light reflection or shine on the surface of fresh highland barley (not glossy—very glossy)
	Grain integrity	Integrity on the surface of of fresh highland barley (incomplete—complete)
	Size uniformity	Size of fresh highland barley (uneven/nonuniform—even/uniform)
Aroma	Cereal aroma	Aroma typical of cereals (none—intensive)
Mouth feel	Hardness	Force required to first bite throughout the sample with the molars (soft—hard)
	Springiness	The elasticity of fresh highland barley that can be stretched and returned to its original length (small—big)
Taste	Overall taste	Overall taste intensity (none—intensive)

**Table 2:** Factors and levels used in orthogonal experimental design.

Level	Tapioca Pearl Optimized Factors		
	A. Tapioca Starch Content (%)	B. Erythritol Stevia Content (%)	C. Cooking Time (min)
1	34	1.5	1.5
2	36	2.0	2.0
3	38	2.5	2.5
Level	Milk Tea BOBA Optimized Factors		
	D. Sodium Alginate Content (%)	E. Calcium Lactate Content (%)	F. Erythritol Stevia Content (%)
4	1.1	0.4	1.8
5	1.3	0.5	2.0
6	1.5	0.6	2.2

**Table 3:** Effects of different treatments on the nutritional composition of fresh highland barley.

Nutrient Composition(g/100 g)	Ash	Lipid	Protein	Total Starch	Total Dietary Fiber
CON	2.28 ± 0.11 ^a^	1.90 ± 0.05 ^c^	9.97 ± 0.75 ^c^	72.59 ± 0.48 ^a^	12.26 ± 0.29 ^b^
UT	1.85 ± 0.03 b	2.24 ± 0.03 ab	11.92 ± 0.13 b	63.70 ± 0.77 b	19.88 ± 0.19 a
MFB	1.58 ± 0.15 d	2.39 + 0.21 a	12.01 ± 0.04 ab	62.44 ± 0.25 bc	19.98 ± 0.04 a
BFB	1.69 ± 0.10 cd	2.31 ± 011 a	12.37 ± 0.15 a	61.81 ± 0.23 c	19.91 ± 0.16 a
SFB	1.71 ± 0.07 c	2.22 ± 0.07 b	12.20 ± 0.21 a	62.14 ± 0.42 bc	20.01 ± 0.56 a
CFB	1.7 ± 0.02 c	2.2 ± 0.12 b	12.06 ± 0.23 ab	61.99 ± 0.37 c	20.01 ± 0.33 a

Each value is expressed as mean ± SD (n = 3). Means with different letters within a row are significantly different (*p* < 0.05). CON, UT, MFB, BFB, SFB, and CFB are maturated highland barley, untreated, microwaved, baked, steamed, and cooked fresh highland barley, respectively.

**Table 4:** The color analysis of fresh highland barley with different treatments.

Treatments	L* (Lightness)	a* (Redness/Greenness)	b* (Yellowness/Blueness)
UT	80.04 ± 0.25 ^a^	1.59 ± 0.11 ^ab^	12.99 ± 0.16 ^ab^
MFB	78.58 ± 0.31 ^ab^	1.64 ± 0.01 ^ab^	13.33 ± 0.06 ^a^
BFB	75.47 ± 0.41 ^c^	1.68 ± 0.51 ^a^	13.41 ± 0.57 ^a^
SFB	79.62 ± 0.12 ^a^	1.61 ± 0.08 ^ab^	13.22 ± 0.50 ^a^
CFB	77.00 ± 0.08 ^b^	1.56 ± 0.04 ^b^	12.49 ± 0.05 ^b^

Each value is expressed as mean ± SD (n = 3). Means with different letters within a row are significantly different (*p* < 0.05). UT, MFB, BFB, SFB, and CFB are untreated, microwaved, baked, steamed, and cooked fresh highland barley, respectively.

**Table 5:** Effects on texture profile of fresh highland barley with different treatments.

Treatments	Hardness/g	Adhesiveness/g	Springiness/mm	Cohesiveness/g	Chewiness/g	Resilience/mm
UT	12413 ± 176 ^a^	4.91 ± 0.27 ^a^	0.31 ± 0.03 ^d^	0.32 ± 0.01 ^d^	4332.81 ± 237.18 ^b^	0.53 ± 0.08 ^a^
MFB	11375.00 ± 364.66 ^b^	2.40 ± 0.33 ^b^	0.76 ± 0.03 ^ab^	0.67 ± 0.02 ^a^	7551.81 ± 441.22 ^a^	0.59 ± 0.05 ^a^
BFB	12452.16 ± 311.26 ^a^	5.24 ± 0.13 ^a^	0.47 ± 0.08 ^c^	0.46 ± 0.03 ^b^	4121.43 ± 170.10 ^b^	0.58 ± 0.01 ^a^
SFB	10988.77 ± 437.37 ^c^	2.38 ± 0.36 ^b^	0.80 ± 0.01 ^a^	0.41 ± 0.03 ^c^	4149.88 ± 243.33 ^b^	0.33 ± 0.02 ^b^
CFB	10594.35 ± 281.56 ^c^	1.39 ± 0.34 ^c^	0.63 ± 0.02 ^b^	0.35 ± 0.02 ^d^	2981.06 ± 287.39 ^c^	0.34 ± 0.03 ^b^

Each value is expressed as mean ± SD (n = 3). Means with different letters within a row are significantly different (*p* < 0.05). UT, MFB, BFB, SFB, and CFB are untreated, microwaved, baked, steamed, and cooked fresh highland barley, respectively.

**Table 6:** The effect of the different treatments on the eGI.

Group	UT	MFB	BFB	SFB	CFB
eGI	60.18 ± 0.25 ^a^	61.12 ± 0.55 ^a^	61.62 ± 0.68 ^a^	58.64 ± 0.74 ^b^	58.72 ± 0.03 ^b^

Each value is expressed as mean ± SD (n = 3). Means with different letters within a row are significantly different (*p* < 0.05). UT, MFB, BFB, SFB, and CFB are untreated, microwaved, baked, steamed, and cooked fresh highland barley, respectively.

**Table 7:** The result of orthogonal test of tapioca pearl formula.

Test Number	Factors			Sensory Score
	A. Tapioca Starch Content/%	B. Erythritol Stevia Content/%	C. Cooking Time/min	
1	1 (34)	1 (1.5)	1 (1.5)	79
2	1	2 (2.0)	2 (2.0)	76
3	1	3 (2.5)	3 (2.5)	82
4	2 (36)	1	2	83
5	2	2	3	86
6	2	3	1	84
7	3 (38)	1	3	87
8	3	2	1	82
9	3	3	2	81
k_1_	79.00	83.00	81.67	
k_2_	84.33	81.33	80.00	
k_3_	83.33	82.33	85.00	
R	5.33	1.67	5.00	
Optimized level	A_2_	B_1_	C_3_	
Influential order	A > C > B			

k_1_, k_2_, k_3_: mean of the values of the test indicators corresponding to the “1,2,3” level; R: range of the values of the test.

**Table 8:** The variance analysis of orthogonal test of tapioca pearl formula.

Factors	SS	df	F-Value	*p*-Value	Sig.
Tapioca starch content	48.222	2	217.216	19.000	**
Erythritol stevia content	4.222	2	19.018	19.000	*
Cooking time	38.889	2	175.176	19.000	**
Error total	0.22	2			

SS: Sum of square; df: Degree of freedom; sig., significant; **: *p* < 0.01; *: *p* < 0.05.

**Table 9:** The result of orthogonal test of milk tea BOBA.

Test Number	Factors			Sensory Score
	D. Sodium Alginate Content/%	E. Calcium Lactate Content/%	F. Erythritol Stevia Content/%	
1	1 (1.1)	1 (0.4)	1 (1.8)	70
2	1	2 (0.5)	2 (2.0)	76
3	1	3 (0.6)	3 (2.2)	80
4	2 (1.3)	1	3	84
5	2	2	1	81
6	2	3	2	85
7	3 (1.5)	1	2	78
8	3	2	3	82
9	3	3	1	79
k_1_	75.33	77.33	76.67	
k_2_	83.33	79.67	79.67	
k_3_	79.67	81.33	82.00	
R	8.00	4.00	5.33	
Optimized level	D_2_	E_3_	F_3_	
Influential order	D > F > E			

k_1_, k_2_, k_3_: mean of the values of the test indicators corresponding to the “1,2,3” level; R: range of the values of the test.

**Table 10:** The variance analysis of orthogonal test of milk tea BOBA.

Factors	SS	df	F-Value	*p*-Value	Sig.
Sodium alginate content	96.222	2	108.236	19.000	**
Calcium lactate content	24.222	2	27.246	19.000	*
Erythritol stevia content	42.889	2	48.244	19.000	*
Error total	0.89	2			

SS: Sum of square; df: Degree of freedom; sig., significant; **: *p* < 0.01; *: *p* < 0.05.

## Data Availability

The original contributions presented in the study are included in the article, further inquiries can be directed to the corresponding author.
